# The Practical Medicine Series of Year-Books

**Published:** 1904-03

**Authors:** 


### Books and Magazines

---

The Practical Medicine Series of Year-Books. Comprising ten volumes on the year's progress in medicine and surgery, under the general editorial charge of Gustavus P. Head, M. D., Professor of Laryngology and Rhinology, Chicago Post-Graduate Medical School.

Before the reviewer is volume II, devoted to general surgery, edited by John B. Murphy, M. D., Professor of Surgery, Northwestern University Medical School. This volume is well illustrated and succinctly gotten up. The price of this volume is $2.00; the entire series, $7.50.

Before the reviewer also is volume III, of the same series, on the eye, ear, nose and throat, edited by C. A. Wood, M. D., A. H. Andrews, M. D., and T. M. Hardie, M. D., of Chicago.

Volume IV, also of the same series, devoted to gynecology, edited by E. C. Dudley, M. D., of Chicago.

These volumes are published by the Year-Book Publishing Company, of 40 Dearborn Street, Chicago. 1903. T. J. B.

